# Inositol Phosphates and Synthesizing Enzymes: Implications in Neurodegenerative Disorders

**DOI:** 10.3390/biom15020225

**Published:** 2025-02-04

**Authors:** Chisom J. Onu, Michael Adu, Mohamed Chakkour, Vikalp Kumar, Miriam L. Greenberg

**Affiliations:** Department of Biological Sciences, Wayne State University, Detroit, MI 48202, USAhi0697@wayne.edu (M.A.); vikalpkumar031@wayne.edu (V.K.)

**Keywords:** inositol, inositol phosphates, psychiatric, neurodegenerative, inositol pyrophosphates

## Abstract

Inositol is a vital sugar molecule involved in numerous signaling pathways required for cellular homeostasis and cell survival. Myo-inositol and its phospho-derivatives, inositol phosphates (IPs), are the most prevalent forms of inositol found in living cells. They are involved in regulating ion channels, metabolic flux, stress response, and other key biological processes. While emerging research has highlighted the significant roles of inositol phosphates in immunity, cancer, and metabolic diseases, there is a lack of comprehensive reviews on their roles in psychiatric and neurological disorders. This review aims to fill that gap by analyzing the existing literature on the importance of inositol phosphates in severe psychiatric and neurological conditions such as Parkinson’s disease, Alzheimer’s disease, bipolar disorder, amyotrophic lateral sclerosis, schizophrenia, and Huntington’s disease, underscoring the potential to pave the way for new treatment regimens for these debilitating disorders targeting inositol pathways.

## 1. Introduction

Inositol is a six-carbon sugar crucial for eukaryotic cell survival. There are nine potential inositol stereoisomers, among which myo-inositol is the most prevalent in living cells [1]. Myo-inositol and its phosphorylated derivatives, inositol phosphates (IPs), are abundant in mammalian cells. These bioactive compounds are involved in various physiological functions, including the regulation of ion-channel permeability, phosphate levels, metabolic flux, gene expression, insulin signaling, embryonic development, and stress response [2]. Alterations in inositol metabolism have been linked to serious medical complications, including anxiety disorders, diabetes, polycystic ovary syndrome, Lowe syndrome, hypercholesterolemia, and cancer development [3,4,5,6].

**Figure 1:**
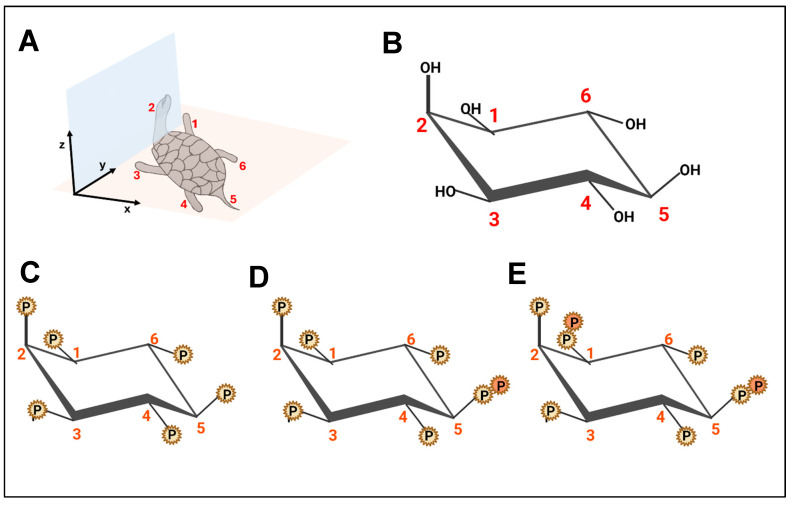
Structural illustration of the (**A**) Agranoff turtle, which is analogous to the thermodynamically stable myo-inositol chair configuration. The turtle’s head represents the hydroxyl group at the C2 position, which is in an axial plane (zy plane), while all 5 other hydroxyl groups represented by the flippers and the tail remain in the equatorial plane (xy plane). (**B**) Chair confirmation of myo-inositol. (**C**) Chair confirmation of inositol hexakisphosphate (IP_6_). (**D**) Chair confirmation of 5-diphosphoinositol pentakisphosphate (5-IP_7_) showing an extra phosphate group attached to the phosphate group at the C5 position. (**E**) Chair confirmation of 1,5-bis-diphospho-inositol tetrakisphosphate (1,5-IP_8_) showing an extra phosphate group attached to the phosphate group at the C5 and C1 positions.

**Figure 2:**
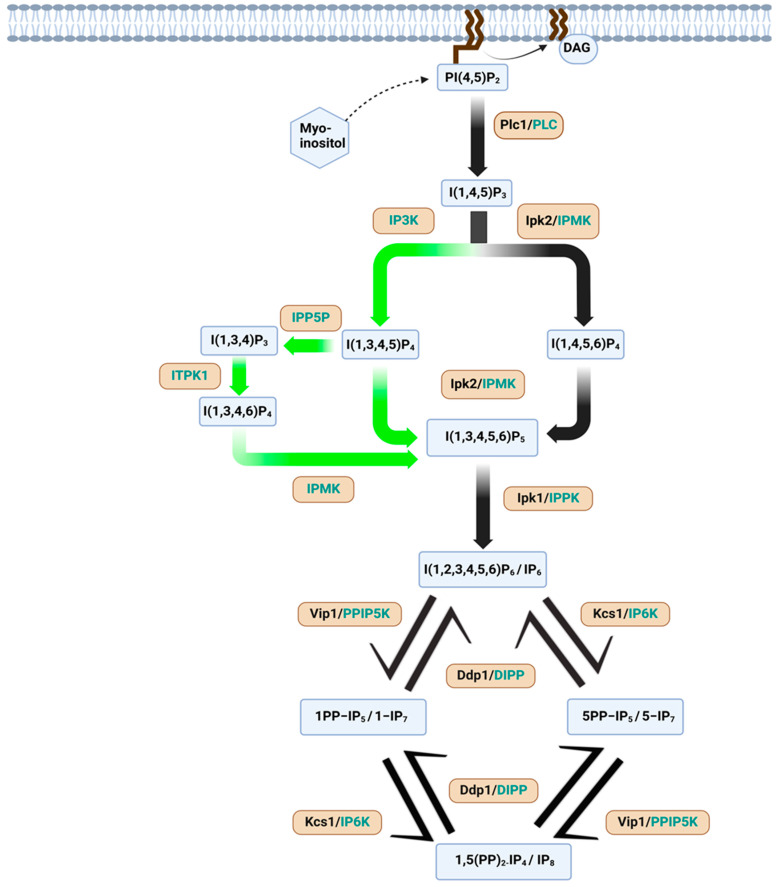
Biosynthetic pathways of inositol phosphates and inositol pyrophosphates. Synthesis of inositol pyrophosphates starts with the generation of IP_3_ from PIP_2_ by the PLC enzyme in mammals and Plc1 in yeast. Mammalian enzymes are represented in the green color, and the arrows in the green color indicate pathways that occur only in mammals. Enzymes represented in the black color are present in yeast, and the black-colored arrows indicate pathways that occur in yeast and mammals.The inositol phosphates are as follows: PI(4,5)P2, phosphatidylinositol 4,5- bisphosphate; DAG, diacylglycerol; I(1,4,5)P3, inositol (1,4,5) trisphosphate; I(1,3,4,5)P4/I(1,4,5,6)P4, inositol tetrakisphosphate; I(1,3,4,5,6)P5, inositol (1,3,4,5,6) pentakisphosphate; I(1,2,3,4,5,6)P6 (IP6), inositol (1,2,3,4,5,6) hexakisphosphate; 1PP-IP5 (1-IP7), 1-diphosphoinositol pentakisphosphate; 5PP-IP5 (5-IP7), 5-diphosphoinositol pentakisphosphate; and 1,5(PP)2-IP4 (IP8), 1,5-bis-diphospho-inositol tetrakisphosphate.The enzymes are as follows: PLC, phospholipase-C; IP3Ks or ITPKs, inositol-trisphosphate 3-kinases; Ipk2, inositol polyphosphate kinase; IPMK, inositol polyphosphate multikinase; IPPK, inositol pentakisphosphate 2-kinase; ITPK1, inositol (1,3,4) trisphosphate 5/6-kinase; PPIP5K, diphosphoinositol pentakisphosphate kinase 1; IP6K, inositol hexakisphosphate kinase; Kcs1, kinase C suppressor 1; DIPP, diphosphoinositol polyphosphate phosphohydrolase; and Ddp1, diadenosine and diphosphoinositol phosphohydrolase.

The biochemistry of myo-inositol and inositol phosphates has been extensively studied due to their physiological importance. Notably, the thermodynamically stable form of myo-inositol that adopts a hydroxyl group at the C2 position is oriented axially, represented as the turtle’s head, while the remaining five hydroxyl groups are positioned equatorially, symbolizing the flippers and tail [Figure 1] [1,7]. In the cell, inositol exists in two distinct forms: as membrane-bound phosphatidylinositol (PI) or as free cytosolic myo-inositol and inositol phosphates [8]. Inositol phosphates can be synthesized by substituting one or more of the myo-inositol hydroxyl groups on the 1–6 positions with phosphate groups [1,9]. Inositol (1,4,5)-trisphosphate (IP3) is produced from phosphatidylinositol 4,5-bisphosphate (PIP2) in a reaction catalyzed by phospholipase C [Figure 2]. Phosphorylation of IP3 yields IP4, IP5, or IP6. Additionally, the hydroxyl groups of inositol may be substituted with diphosphates, generating inositol pyrophosphates. Diphosphoinositol pentakisphosphate (IP7) and bisdiphosphoinositol tetrakisphosphate (IP8) are members of the inositol pyrophosphate family [Figure 2] [1]. By virtue of their high-energy phosphoanhydride bonds, inositol pyrophosphates engage in a variety of cellular and signaling processes. They can either bind specific proteins or transfer their β-phosphate to pre-phosphorylated serine residues, resulting in a novel post-translational modification known as serine pyrophosphorylation [10]. Inositol hexakisphosphate kinases (IP6Ks) phosphorylate IP6 to IP7 and IP8. Three isoforms of IP6K exist in humans, namely IP6K1, IP6K2, and IP6K3 [11]. Among these, IP6K1 and IP6K2 are found in various tissues [12], while IP6K3 shows substantial expression in the cerebellum [13].

**Figure 3:**
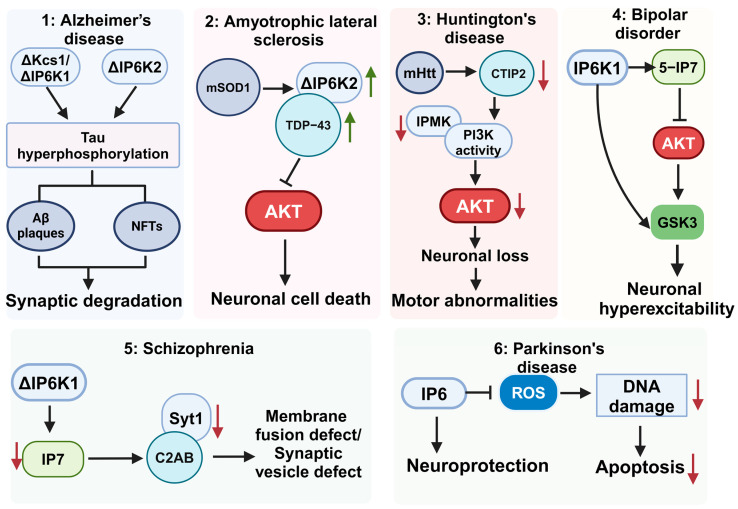
Inositol phosphates and synthesizing enzyme signaling in neurological disorders. (**1**) Kcs1 and IP6K2 depletion causes tau hyperphosphorylation, resulting in Aβ plaques and synaptic dysregulation. (**2**) IP6K2 interacts with TDP-43, causing cellular stress, Akt inhibition, and neuronal cell death. (**3**) CTIP2 is dysregulated in HD. CTIP2 regulates IPMK levels, and impaired IPMK levels lead to decreased PI3K activity and PIP3 levels. Diminished PIP3 levels decrease Akt activity and neuronal death. (**4**) IP6K1 synthesizes IP7 which inhibits Akt and indirectly activates GSK3 resulting in neuronal hyperexcitability. IP6K1 also binds directly to GSK3 to activate it and increase neuronal excitability. (**5**) IP6K2 regulates Syt1 via its product IP7 that binds to C2AB domain of Syt1. The IP7 interaction with Syt1 disrupts the recycling of synaptic vesicles and the release of neurotransmitters. (**6**) IP6 is a neuroprotectant that decreases PD’s ROS, DNA damage, and apoptosis. Kcs1, inositol hexakisphosphate/heptakisposphate kinase; IP6K1, inositol hexakisphosphate kinase 1; CTIP2, COUP-TF-interacting protein 2; IPMK, inositol-polyphosphate multikinase; IP3K, inositol-trisphosphate 3-kinases; ROS, reactive oxygen species; IP6, inositol hexaphosphate; TDP-43, TAR DNA-binding protein 43. Black arrows represent direction; red: decrease; green: increase.

In the past decade, several review articles have summarized the roles of inositol phosphates and pyrophosphates in health, disease progression, and prevention. Inositol and its derivatives have been shown to play significant biological roles in immunity [14], cancer development [15], the prevention of colitis-induced carcinogenesis [16], and the enhancement of the anticancer potency of conventional chemotherapy [17]. The enzymes involved in the synthesis of inositol pyrophosphates have been implicated in health, metabolic disease progression, and cancer [18,19,20]. The current review focuses on the roles of inositol phosphates in psychiatric and neurodegenerative diseases, including bipolar disorder (BD), schizophrenia, Alzheimer’s disease (AD), amyotrophic lateral sclerosis (ALS), Parkinson’s disease (PD), and Huntington’s disease (HD) summarized in Table 1 and Figure 3.

## 2. Inositol Phosphates and Their Synthesizing Enzymes in Psychiatric Diseases

Inositol phosphates (IPs) play a critical role in various processes associated with psychiatric illnesses, including mood regulation, neurotransmitter signaling, and synaptic plasticity. Consistent with the vital roles played by IPs, IP synthesizing enzymes such as IP6K1, IP6K2, IP6K3, and IPK2 are highly expressed in neuronal cells, suggesting that they play crucial roles in neurons and, by extension, neurodegenerative and psychiatric diseases [31]. Dysregulation of IPs and the enzymes that synthesize them can lead to neuronal dysfunction, a common hallmark in both psychiatric and neurodegenerative diseases. One of the key contributors to neuronal dysfunction is the malfunction of Ca^2+^ pumps, which are regulated by IPs such as PIP2 and IP3.

IP3 plays a crucial role in steering axonal growth cones during neural development. In addition, brief elevations in intracellular Ca^2+^ levels, known as Ca^2+^ transients, determine whether a neuron will be excitatory or inhibitory by regulating the release of neurotransmitters. For instance, low-frequency Ca^2+^ oscillations promote the release of excitatory neurotransmitters like acetylcholine and glutamate, while higher-frequency oscillations lead to the release of inhibitory neurotransmitters such as glycine and γ-aminobutyric acid (GABA) [32].

These rhythmic oscillations are essential for normal brain functions such as sleep, memory, and learning [33]. However, distortions in IP3 and Ca^2+^ signaling can disrupt these processes, leading to brain disorders such as AD, autism, epilepsy, and schizophrenia [33,34,35]. In this section, we discuss the roles of inositol phosphates and their synthesizing enzymes in neurodegenerative and psychiatric diseases.

### 2.1. Alzheimer’s Disease (AD)

AD is characterized by the accumulation of β amyloid (Aβ) plaques and neurofibrillary tangles (NFTs), which are associated with tau protein hyperphosphorylation [21]. Tau is a neuronal protein associated with microtubules and helps stabilize the microtubule complex. In AD, tau proteins become highly phosphorylated and stick to each other, blocking neuronal transport and impairing synaptic communication [36]. In humanized yeast cells expressing tau proteins, deletion of Kcs1, the yeast homolog of IP6K1, increased tau hyperphosphorylation [21], suggesting a role for IP6K1 in preventing tau hyperphosphorylation. The mechanisms by which Kcs1 prevents the hyperphosphorylation of tau proteins remain unclear, highlighting an intriguing avenue for future molecular investigations. In addition, whether the human IP6K1 performs a similar function remains to be investigated. Interestingly, critical enzymes in the IP pathway such as IPK2, which converts IP3 to IP5, are also implicated in increased tau hyperphosphorylation [21]. IPK2 deletion resulted in increased hyperphosphorylation of tau proteins [21]. These outcomes suggest a significant role for inositol phosphates in AD pathophysiology. Another IP kinase, IP6K3, is highly enriched in neurons. Studies have identified two single nucleotide polymorphisms (SNP) in the promoter region of IP6K3 associated with sporadic AD [22]. One of the SNPs characterized by a luciferase assay showed increased promoter activity consistent with increased IP6K3 expression [22].

The accumulation of amyloid beta peptide (Aβ) oligomers, misfolded products of amyloid precursor protein (APP), is a hallmark of AD onset. Aβ accumulation has been shown to impair IP3 signaling and calcium (Ca^2+^) homeostasis [37]. Dysregulation of IP3 and Ca^2+^ is evident in tau hyperphosphorylation, reactive oxygen species (ROS) production, and synaptic plasticity impairment [37]. Aβ aggregates hyperactivate IP3 receptors (IP3R) and ryanodine receptors (RyR), leading to excessive Ca^2+^ release from the endoplasmic reticulum (ER) and disrupting Ca^2+^ homeostasis [38,39]. In neurons, this cascade leads to loss of dendritic spines, synaptic degradation, and neuronal cell death [40], all of which can be manifested in long-term depression [37]. Elevated levels of Ca^2+^ also lead to hyperactivity of the phosphatase calcineurin (CaN), which could manifest as memory impairment, neuroinflammation, hyperphosphorylated tau, and neuronal death [41]. Thus, the IP3/Ca^2+^ signaling pathway plays a critical role in neuronal function. Studies have shown that IP6 enhances the removal of Aβ by blocking β-site amyloid precursor protein cleaving enzyme 1 (BACE1), a beta-secretase enzyme that cleaves APP into Aβ. Inhibition of BACE1 by IP6 reduces Aβ levels [42,43]. In addition, IP6 activates autophagy by regulating the expression of autophagy-related proteins such as berlin-1, LC3B, and sirtuin 1 (SIRT1) [44]. SIRT1 has been shown to regulate autophagy by deacetylating key autophagy molecules such as FOXO3, Beclin-1, ATGs, and LC3B. Autophagy regulates Aβ generation and clearance, and increased autophagy can ameliorate the deleterious effects of Aβ accumulation. These studies suggest that Aβ accumulation in AD disrupts IP3 receptors, leading to dysregulation of Ca^2+^ homeostasis and neuronal death.

### 2.2. Amyotrophic Lateral Sclerosis (ALS)

ALS is a fatal neurodegenerative disease that affects motor neurons responsible for controlling voluntary muscles [23]. The exact cause of ALS remains unknown, but a combination of genetic, environmental, and aging factors has been implicated in its development. About 90% of ALS cases are sporadic, with no clear family history, while the remaining 10% are familial. One of the first genetic mutations identified in familial ALS is a mutation of the superoxide dismutase 1 (SOD1) gene [23]. Mutations in SOD1, along with models such as induced pluripotent stem cells (iPSCs), G93ASOD1 transgenic mice (TG), and neuronal cell lines such as SHSY-5Y, have been widely used to study ALS pathogenesis [11]. Recent studies have pointed to the involvement of the IP synthesizing enzyme IP6K2 in ALS onset. Notably, IP6K2 mRNA and protein levels increase in mutant SOD1 transgenic mice prior to ALS symptom onset, suggesting that IP6K2 could serve as an early, presymptomatic marker of the disease [11]. Activation of this enzyme in the cytoplasm coincides with ALS progression, suggesting that it plays a role in the early stages of the disease.

Another important aspect of ALS pathology is the accumulation of TAR DNA-binding protein 43 (TDP-43) in the cytoplasm. Under normal conditions, TDP-43 regulates exon splicing and stabilizes mRNA in the nucleus. However, in ALS, it aggregates in the cytoplasm, forming toxic inclusions—a hallmark of the disease. The accumulation of TDP-43 triggers a cellular stress response, which in turn activates IP6K2 and promotes its cytoplasmic translocation [23]. In the cytoplasm, TDP-43 interacts with IP6K2, resulting in neuronal cell death. This process involves the downregulation of Akt, a kinase crucial for cell survival. Reduced phosphorylation of Akt in SH-SY5Y neuronal cells results in apoptosis, further contributing to motor neuron degeneration in ALS [23].

Recent studies have further implicated IP6K2 in ALS. Using liquid chromatography–mass spectrometry (LC-MS), Ito et al. observed elevated levels of IP7, a metabolite synthesized by IP6K2, in the spinal cords of SOD1(G93A) transgenic mice in the late stages of ALS, as well as in postmortem ALS patient samples [24]. This increase in IP7 and the IP7/IP6 ratio suggests heightened activity of IP7-synthesizing kinases, such as IP6K2. The study also found that IP6K2 interacts with phosphorylated TDP-43, contributing to cytoplasmic inclusion formation and neuronal cell death in ALS patients [24].

Overall, these findings underscore the crucial role of IP6K2 in ALS pathology, particularly in TDP-43-mediated neurodegeneration. Increased IP6K2 expression and cytoplasmic translocation before ALS onset suggest that IP6K2 may play a role in ALS onset. Whether this increase in IP6K2 activity represents a neuronal response to counteract neurodegeneration or serves as an indicator or direct cause of neurodegeneration remains unclear. In addition, it is unknown whether other IP6K homologs (IP6K3 and IP6K1) contribute to the increased IP7 pool, an interesting question to probe in future studies.

### 2.3. Huntington Disease (HD)

HD is a neuronal disorder that affects the striatum in the forebrain and is characterized by motor abnormalities and the expansion of glutamine repeats in the mutant huntingtin (mHtt) gene [25]. Inositol phosphate synthesizing enzymes have been implicated in HD pathology. Among these, levels of inositol polyphosphate multikinase (IPMK) are reduced in HD patients and in mouse models of HD [25]. IPMK converts IP3 to IP5 and possesses PI3K activity, which converts PIP2 to PIP3. IPMK expression is regulated by chicken ovalbumin upstream promoter transcription factor (COUP-TF)-interacting protein 2 (CTIP2), which is enriched in the striata and impaired by mutant huntingtin proteins [25]. IPMK mRNA and protein levels were found to be significantly reduced in mouse and cell models of HD [25]. The overexpression of CTIP2 in a striata cell line model of HD characterized by 111 glutamine repeats restored IPMK protein levels to those of the control [25]. Decreased IPMK levels in HD patients leads to decreased PI3K activity and PIP3 levels [25,45,46,47]. Because PIP3 activates Akt, Akt activity is reduced as well. Akt is a serine/threonine protein kinase involved in regulating several signaling pathways, such as cell proliferation, survival, and metabolism. Akt plays a crucial role in protecting and preserving the health of neuronal cells, and its decreased activity results in increased neuronal loss [25], suggesting that the PI3K activity of IPMK is necessary for the survival of striatal neurons [25].

Interestingly, while Htts affects IPMK via decreased CTIP2 expression, IPMK also affects Htts [25]. The administration of an IPMK-expressing adenovirus serotype into the striatum in an Htt mouse model decreased the abundance and size of Htt aggregates by 75% and 30%, respectively, resulting in improved central locomotive activity [25]. This suggests that the accumulation of Htt aggregates in HD is a consequence of decreased IPMK.

Impaired oxidative phosphorylation due to decreased complex I and IV activities has previously been reported in HD patients [48]. Interestingly, IPMK restored oxidative phosphorylation in the striatal-derived Htt cell line model (Q111). IPMK possesses both inositol phosphate kinase and PI3 kinase activities. To determine which activities of the IPMK protein are responsible for restoring metabolic defects in the HD cell model, researchers mutated the kinase domain (amino acids 129–235) to alanine to create an IPMK-kinase dead protein, which lacks both PI3K and IP kinase activities [25]. They also utilized atIPK2β, a plant-derived IPMK ortholog from Arabinose thaliana, which has IP kinase activity and lacks PI3K activity. Neither the IPMK-kinase dead nor atIPK2β was able to rescue the decreased cell viability and proliferation of Q111 cells, suggesting that the PI3-kinase activity of IPMK is essential for metabolic rescue in the HD cell model.

In contrast to IPMK, levels of which are reduced in HD, IP6K2 is activated in HD patients. IP6K2 translocates from the nucleus to the cytoplasm when activated. Increased cytoplasmic translocation of IP6K2 and increased IP7 levels have been observed in HD patients [23,26].

### 2.4. Bipolar Disorder (BD)

BD is a recurrent psychiatric illness that affects about 2% of the global population [49]. It is characterized by mood swings between mania and depression, along with changes in energy levels, cognition, and behavior [47,50,51]. Lithium, the first approved treatment for BD, remains one of the most commonly prescribed mood stabilizers [52,53]. The discovery that lithium depletes cellular inositol led to the inositol depletion hypothesis, proposed by Berridge and colleagues, which suggests that mood stabilizers reduce excessive inositol triphosphate (IP3)-induced Ca^2+^ release from mitochondria in hyperexcitable neurons, a dysfunction thought to underlie BD [54,55,56].

The inositol depletion hypothesis is further supported by evidence that structurally different mood stabilizers, such as valproate (VPA) and carbamazepine, also reduce inositol levels [56,57,58]. The first and rate-limiting step in inositol synthesis is the conversion of glucose-6-phosphate to inositol-3-phosphate, a reaction catalyzed by myo-inositol-3-phosphate synthase (MIPS), which is dephosphorylated by inositol monophosphatase (IMPase) to yield inositol, reviewed by [56]. Lithium uncompetitively inhibits IMPase by displacing its Mg^2+^ cofactors, reducing inositol levels [59,60]. In contrast, VPA does not affect IMPase but decreases inositol synthesis by indirectly inhibiting MIPS [61,62,63]. In mouse embryonic fibroblasts, VPA decreases inositol levels via IP6K1-mediated inhibition of ISYNA1, the gene encoding MIPS [64,65].

IP6K1 plays a broader role in mammalian cells by regulating various physiological processes through the production of the highly energetic inositol pyrophosphate 5-IP7 [10]. Although it is not fully understood whether the inhibition of ISYNA1 by IP6K1 is mediated by 5-IP7, the enzyme has been linked to psychiatric disorders such as BD. IP6K1-generated 5-IP7 inhibits Akt, which in turn leads to increased glycogen synthase kinase 3 (GSK3) activity [66]. Reduced Akt activity and elevated GSK3 activity are associated with BD, contributing to neuronal hyperexcitability [67,68].

The inhibition of GSK3 by lithium is thought to be a key therapeutic mechanism in BD, as it helps modulate neuronal excitability [69]. Interestingly, studies in mice show that the deletion of IP6K1 leads to reduced GSK3 activity in the brain, producing behavioral changes similar to those observed in GSK3 mutant mice. This suggests that targeting the IP6K1–GSK3β interaction could offer therapeutic benefits for BD and other psychiatric disorders involving GSK3 dysregulation [65].

In summary, the modulation of inositol metabolism and the Akt-GSK3 pathway appear to be critical in understanding and treating BD, with lithium and other mood stabilizers exerting their effects through these pathways. Targeting key regulators such as IP6K1 could open new avenues for BD therapeutics.

### 2.5. Schizophrenia

Schizophrenia is a psychiatric disorder characterized by disruptions in thought processes, social interactions, and perceptions. The disruption of synaptic vesicle cycling, which is necessary for neurotransmitter release at synapses, is one of the hallmarks of schizophrenia [70,71]. Central to this dysregulation are proteins involved in presynaptic vesicle exocytosis, such as synaptotagmin 1 (Syt1), proteins that mediate the release of neurotransmitters [72].

Synaptic vesicle cycling is a multistep process that releases neurotransmitters from tiny organelles called synaptic vesicles. These organelles fuse with plasma membranes to release their content with the aid of a calcium sensor, Syt1, and soluble N-ethylmaleimide-sensitive factor attachment protein receptor (SNARE) proteins. The release of neurotransmitters between pre- and postsynaptic neurons leads to the activation or inhibition of the postsynaptic compartment and plays a crucial role in neuron-to-neuron communication.

IP6K1 regulates synaptotagmin 1 (Syt1), a critical player in the synaptic vesicle cycling sensor essential for neurotransmitter release [27]. IP6K1-produced IP7 binds to the C2AB domain of Syt1, which senses Ca^2+^ and mediates membrane fusion [27]. The suppression of Syt1 inhibits synaptic vesicle exocytosis and represses Syt1 [27]. Interestingly, hippocampal slices from IP6K1 knockout mice exhibit increased presynaptic release and exocytosis [73]. Moreover, the catalytically inactive form of IP6K1 can partially rescue synaptic deficits in these knockout mice, suggesting that IP6K1 has both IP7-dependent and independent functions in synaptic vesicle trafficking.

In summary, IP6K1 is a crucial regulator of synaptic vesicle cycling. The dysregulation of synaptic vesicle cycling leads to synaptic dysfunction and neurotransmitter imbalances, contributing to the pathogenesis of schizophrenia.

### 2.6. Parkinson Disease (PD)

PD is a progressive neurodegenerative disorder that affects dopamine-producing (dopaminergic) neurons in the substantia located in the basal ganglia of the brain. Dopamine aids in movement coordination, and a decline in the number of dopaminergic neurons results in uncoordinated movements, such as shaking and difficulty with coordination and balance, characteristic features of PD.

Studies using postmortem brains have shown iron accumulation in the substantia nigra, suggesting a role of iron in PD [74]. Iron has been implicated in the onset of PD pathology [47,75,76]. Excess iron in brain cells reacts with hydrogen peroxide via the Fenton reaction, resulting in the generation of free radicals [75,76]. When produced excessively, these free radicals overwhelm the cell’s antioxidant capabilities, causing oxidative stress that damages DNA, oxidizes lipids, impairs mitochondrial function, and disrupts protein folding. Ultimately, this can lead to the death of neurons. This type of damage has been linked to the development of various neurodegenerative diseases, including PD [75,76,77]. Studies have shown that iron can interact with dopamine and promote its oxidation to form neurotoxic compounds that damage neurons and lead to the characteristic loss of dopaminergic neurons in PD [78,79].

Sabin reported that symptoms in early- and late-onset PD patients improved after IP6 oral administration [80]. Animal and cell experiments revealed that neurons were protected by IP6 in PD [81,82]. Several studies have reported that IP6 inhibits the formation of reactive oxygen species (ROS) and protects cells from oxidative damage [28]. IP6 prevented the DNA damage and cell apoptosis induced by iron in N27 dopaminergic neurons [29].

Zhang et al. showed that IP6 inhibited apoptosis in PD cellular models by blocking the elevation of intracellular calcium levels and subsequently inhibiting the calcium-induced aggregation of α-synuclein [30]. In a cell culture model, Xu et al. reported that IP6 protected dopaminergic neurons against 1-methyl-4-phenylpyridinium (MPP+)-induced apoptosis, even in iron-excess conditions. MPP+ is an inhibitor of oxidative phosphorylation and is derived from the neurotoxin 1-Methyl-4-phenyl1-1,2,3,6-tetrahydropyridine (MPTP) and used to simulate PD disease models [83]. This suggests a neuroprotective effect of IP6 in slowing neuronal cell death in PD due to its antioxidant property and iron-chelating ability.

Although IP6 plays a neuroprotective role in PD, the mechanism underlying its protective role has not been extensively studied. Zhang et al. argued that decreased α-synuclein aggregation and calcium could be a potential mechanism by which IP6 confers neuroprotection [30]. However, whether decreased α-synuclein accumulation is a consequence of reduced calcium levels by IP remains unanswered.

## 3. Conclusions

Inositol phosphates and their synthesizing enzymes are integral to numerous physiological functions [Figure 3]. They serve as essential regulators in the development of the nervous system and play a critical role in maintaining neuronal functions. For example, IP3 facilitates the release of Ca^2+^ from the endoplasmic reticulum, which is essential for controlling axonal growth cone dynamics. Similarly, IP7 plays a significant role in regulating synaptic vesicle trafficking and neurotransmitter release at synapses. We are just starting to understand the role of inositol phosphates and their synthesizing enzymes in neurodegenerative and psychiatric diseases, and many important questions are yet to be answered. As an example, understanding the role of IP6K1 in tau protein phosphorylation may shed light on the molecular events leading to the aggregation of Aβ plaques and NFTs in AD. Further, elucidating the molecular pathways that result in elevated mRNA and protein levels of IP6K2 may provide deeper insight into the onset of ALS. Moreover, it will be interesting to determine how IP6K1/IP7 mediates synaptic vesicle trafficking in neurons, and this may address the dysregulation of synaptic vesicle cycling in schizophrenia. Deciphering molecular mechanisms underlying IP6’s neuroprotective function in PD will provide a deeper understanding of dysregulated neurological pathways in the diseased condition. Advancing our knowledge by answering these unresolved questions could open new avenues for treatment regimens for these debilitating disorders.

## Figures and Tables

**Table 1:** Summary of the roles of inositol phosphates and their synthesizing enzymes in neurodegenerative disorders.

Disease Name	Inositol Phosphate/Inositol Synthesizing Enzyme	Sense of Change	References
Alzheimer Disease	Kcs1/IP6K1	Decrease	[21]
	IPK2	Decrease	[21]
	IP6K3	Increase	[22]
Amyotrophic lateral sclerosis	IP6K2	Increase	[11,23,24]
Huntington disease	IPMK	Decrease	[25]
	IP6K2	Increase	[23,26]
Schizophrenia	IP6K1	Decrease	[27]
Parkinson disease	IP6	Decrease	[28,29,30]
Bipolar disorder	IP6K1	Increase	

## Data Availability

All data are contained within the manuscript.
